# Treatment outcomes in non-occlusive mesenteric ischemia and post-treatment return to social activities

**DOI:** 10.1007/s00595-024-02909-8

**Published:** 2024-08-01

**Authors:** Gaku Ohira, Koichi Hayano, Toru Tochigi, Tetsuro Maruyama, Takeshi Toyozumi, Yoshihiro Kurata, Michihiro Maruyama, Satoko Arai, Taka-Aki Nakada, Hisahiro Matsubara

**Affiliations:** 1 Department of Frontier Surgery, Chiba University Graduate School of Medicine, 1-8-1 Inohana, Chuuou-Ku, Chiba, 260-8670 Japan; 2 Department of Emergency and Critical Care Medicine, Chiba University Graduate School of Medicine, 1-8-1 Inohana, Chuuou-Ku, Chiba, 260-8670 Japan

**Keywords:** Non-occlusive mesenteric ischemia, Portal venous gas, Return to social activities

## Abstract

**Purpose:**

To investigate the treatment outcomes of patients with non-occlusive mesenteric ischemia (NOMI) at our institution, we focused on their post-treatment return to social activities.

**Methods:**

This study included patients with suspected NOMI who were referred to our department between 2011 and 2023. In-hospital mortality was also investigated as a prognostic factor. The Glasgow–Pittsburgh Outcome Categories (GPOC) score was used to evaluate the return to social activities. The relationship between in-hospital mortality and GPOC scores and patient background and treatment factors was examined.

**Results:**

Eighty-two patients were included in the study. Among them, 54 (65.9%) died during hospitalization. Only 9 patients (11%) returned to their social activities. In the multivariate analysis, non-surgical management was found to be the only independent factor for in-hospital mortality. Positive portal venous gas on computed tomography, no open abdomen, no pre-onset catecholamine administration, platelet count < 100,000/µL, lactate level < 5 mmol/L, APTT < 46 s, and Sequential Organ Failure Assessment score < 11 were factors significantly associated with an increased likelihood of return to social activities.

**Conclusion:**

This is the first study to assess the post-treatment return to social activities among patients with NOMI. Our findings highlight the concerning reality that survivors may face prolonged dependence on medical care.

## Introduction

Non-occlusive mesenteric ischemia (NOMI) occurs when blood supply to the mesentery is reduced to prioritize vital organs, including the brain and heart, during hypovolemia [1]. This phenomenon is a key element in the fight-or-flight response, as proposed by Canon [2]. Although numerous studies have reported NOMI outcomes, most have focused on the survival, hospital discharge, or hospital transfer as endpoints [3–8]. However, clinical practice shows that even surviving patients face significant consequences in their daily lives, with only a few achieving adequate return to social activities [9]. Given the limited data, the recovery rates in this cohort remain unclear. NOMI treatment requires substantial medical resources, including perioperative intensive care and multiple surgeries. If a patient does not return to social activities after treatment, ongoing medical resources are needed. Therefore, evaluating the status of social reintegration after treatment is crucial for assessing cost-effectiveness and planning future treatment strategies.

To address this gap, the present study investigated the treatment outcomes of patients with NOMI at our institution, focusing particularly on their post-treatment return to social activities.

## Materials and methods

This study included patients with suspected NOMI, who were referred to our department between January 2011 and December 2023. NOMI was diagnosed based on positive findings of intestinal ischemia without obstruction of the main mesenteric vessels on intraoperative assessment or contrast-enhanced computed tomography (CT). Both preoperative CT and intraoperative diagnoses were made by a gastrointestinal surgeon with over 10 years’ experience in managing abdominal emergencies. Abdominal angiography was not routinely performed for a NOMI diagnosis; it was only indicated in cases that required arterial injection therapy. Patients with intestinal necrosis were considered to require surgery. Patients without necrosis, considered to be in the ischemic stage, were treated with arterial injection therapy. The final treatment plan was determined based on these principles, the patient’s general condition, and the decisions of the patient and family.

Data on patient background, routine blood tests, Sequential Organ Failure Assessment (SOFA) scores, CT findings, comorbidities, treatment details, resected bowel lengths (if surgery was performed), open abdomen status, and need for second-look surgery were collected. In-hospital mortality was investigated as a prognostic factor and its relationship with patient background and treatment factors was examined. Blood and vital sign data were obtained at the time of NOMI diagnosis. The Glasgow–Pittsburgh Outcome Categories (GPOC) (Table 1) [10] score, comprising the Cerebral Performance Category (CPC) and Overall Performance Category (OPC), was used to evaluate the return to social activities, with scores of 1–2 indicating a good prognosis. In this study, return to social activities was defined as CPC 0 or 1 and OPC 0 or 1, based on a previously established definition [11]. In this state, patients can perform daily activities without assistance, which implies that they do not require investment of medical resources. The medical records of patients with follow-up periods exceeding 28 days were assessed to determine the best GPOC score. Subsequently, the relationship between the GPOC scores, patient backgrounds, and treatment factors was examined.

**Table 1:** Glasgow–Pittsburgh Outcome Categories

	CPC	OPC
1	Clear consciousness, able to lead a normal life, and able to work	CPC 1 and mild disability from non-brain causes
2	Conscious. Able to work part time in a protected situation, able to perform daily activities such as dressing, traveling, cooking, etc., without assistance	CPC 2 and moderate disability due to a cause other than the brain, or a combination of both
3	Conscious. Needs assistance in daily living due to brain damage. At least cognitive ability is impaired	CPC 3 or severe disability due to causes other than the brain, or a combination of both. Requires assistance in daily living
4	Coma, vegetative state	Same as CPC 4
5	Death	Same as CPC 5

*CPC* cerebral performance categories, *OPC* overall performance categories

We used the Chi-squared and Fisher’s exact tests to compare clinical outcomes between groups, as appropriate for categorical variables. Continuous variables, such as age and laboratory data, were categorized into two groups based on the median values for the entire cohort for the analyses. A logistic regression analysis was used for the multivariate analysis. Statistical significance was set at *P* < 0.05. Data were statistically analyzed using the JMP software program (version 16.2; SAS Institute Inc., Cary, NC, USA).

This study was approved by the Ethics Committee of our hospital. Due to its retrospective nature, written informed consent was substituted with a publicly posted disclosure document with an opt-out option.

## Results

**Fig. 1:**
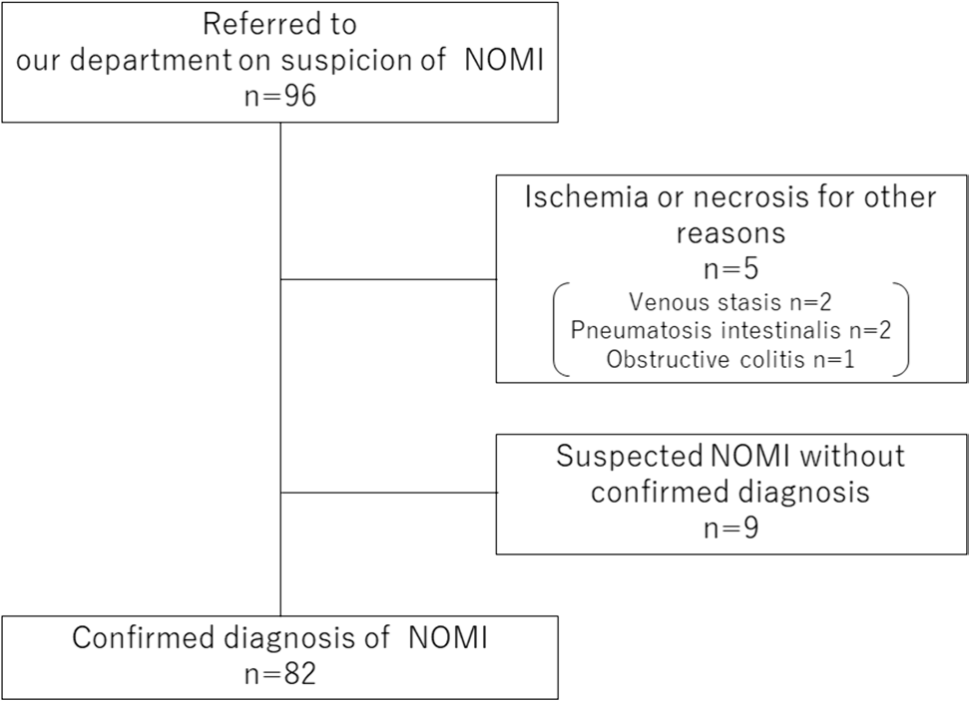
Flowchart. Overall, 96 patients with suspected non-occlusive mesenteric ischemia (NOMI) consulted our department during the study period. Five patients with intestinal ischemia from other causes and nine without a definitive diagnosis were excluded. Ultimately, 82 patients were included in the study

Overall, 96 patients with suspected NOMI consulted our department during the study period. However, five patients with intestinal ischemia from other causes and nine patients without a definitive diagnosis were excluded (Fig. 1). Ultimately, 82 patients were included in the study.

Patient background data are summarized in Table 2. Of the 82 patients diagnosed with NOMI, 10 had only non-enhanced CT images because contrast imaging could not be performed. Among the 72 patients with contrast-enhanced CT, NOMI could not be ruled out, leading to an intraoperative diagnosis of NOMI in 21 patients. Overall, 31 patients (37.8%) were diagnosed based on intraoperative findings, and 59 (72.0%) underwent surgery. One patient who underwent surgery was initially judged to be in the ischemic stage, and intra-arterial vasodilator therapy was initiated; however, the patient’s condition deteriorated, and surgery was performed the next day. Of the 23 patients who did not undergo surgery, 22 required surgery because intestinal necrosis could not be excluded. However, their condition was deemed too critical to benefit from the surgery. Only one patient was judged to be in the ischemic stage and was successfully treated with intra-arterial vasodilator therapy. Among the 22 patients who were deemed too critically ill, two were treated palliatively with intra-arterial vasodilator therapy. Papaverine hydrochloride was administered to all four patients who underwent intra-arterial vasodilator therapy through the superior mesenteric artery immediately after the decision. One patient who underwent surgery received a continuous infusion (60 mg/h) for 3 days until the second-look surgery. One of the 3 non-operative patients received only 1 shot of papaverine 40 mg, 1 received 60 mg/h for 4 days until death, and 1 received 86 mg/h for 3 days until his general condition improved.

**Table 2:** Demographic and clinical characteristics

Categories	Median/no. (%)
Gender	
Male	53 (64.6)
Female	29 (35.4)
Age (years)	74.5 (16–90)
Comorbidities	
Diabetes	17 (20.7)
Hypertension	41 (50)
Hyperlipidemia	17 (20.7)
Heart disease	37 (45.1)
History of cardiovascular surgery or intervention	31 (37.8)
Renal dysfunction	66 (80.5)
Chronic renal replacement therapy	6 (7.3)
History of cardiopulmonary arrest	15 (18.3)
Pre-onset catecholamine administration	38 (46.3)
Pre-onset norepinephrine dose > 0.1 μg/kg/min	21 (25.6)
Pre-onset blood purification therapy	42 (51.2)
Continuous hemodialysis and filtration	41 (50.0)
Extracorporeal membrane oxygenation	4 (4.9)
Diagnosis procedure	
Contrast-enhanced CT findings	51 (62.3)
Intra-operative findings	31 (37.8)
Laparotomy	
Yes	59 (72.0)
No	23 (28.0)

After the diagnosis, blood purification therapy was performed in 52 patients (63.4%). Among the 52 patients, 47 underwent continuous hemodialysis and filtration, 1 patient underwent extracorporeal membrane oxygenation, and 5 patients underwent both procedures. No patient received Polymyxin B hemoperfusion therapy in this study.

The median survival time was 27 days, with 54 (65.9%) patients succumbing to hospitalization. Of the 40 (48.8%) patients who survived for 28 days, only 9 (11%) returned to social activities (CPC or OPC scores of 1–2) (Table 3).

**Table 3:** Glasgow–Pittsburgh Outcome Categories of patients who survived over 28 days (n = 40)

(No.)		Cerebral performance categories			
		1	2	3	4
Overall performance categories	1	2	1	0	0
	2	0	6	0	0
	3	0	4	22	0
	4	0	0	0	7

Factors affecting in-hospital mortality are presented in Table 4. Among those who died during hospitalization, a significantly shorter survival time was associated with the following factors: absence of portal venous gas on CT, no surgery, resected bowel length ≥ 160 cm, pre-onset catecholamine administration, pre-onset blood purification therapy, and pre-onset norepinephrine administration (≥ 0.1 γ). Routine blood tests showed that platelet count < 100,000/µL, lactate level > 5 mmol/L, APTT > 46 s, and SOFA score > 11 were common risk factors for in-hospital mortality. Furthermore, a multivariate analysis was performed in cases with low *P*-values, revealing that non-surgical management was the only independent factor of in-hospital mortality.

**Table 4:** Demographics associate with in-hospital mortality

		In-hospital death	Discharge/transfer	Univariate analysis (*P* value)	Multivariate analysis		
					Odd ratio	95%CI	*P* value
Gender	Male	35	18	1.0			
	Female	19	10				
Age (years)	< 75	24	17	0.1623			
	≥ 75	30	11				
BMI (kg/m^2^)	< 21	25	9	0.1599			
	≥ 21	25	18				
Diagnosis procedure	CECT	33	19	0.6327			
	Intra-operative findings	21	9				
CECT findings							
Defect of intestinal wall enhancement	( +)	45	22	1.0			
	(−)	3	2				
Pneumatosis intestinalis	( +)	26	17	0.2360			
	(−)	27	10				
Ascites	( +)	35	13	0.1225			
	(−)	18	14				
Free air	( +)	10	3	0.7440			
	(−)	42	21				
Hepatic portal venous gas	( +)	9	12	0.0083			
	(−)	44	15		2.50	0.56–12.1	0.2304
Laparotomy	( +)	34	25	0.0183	8.77	1.70–71.5	0.0078
	(−)	20	3				
Open abdomen	( +)	20	10	0.1923			
	(−)	14	15				
Length of resected small bowel	< 110 cm	13	14	0.1759			
	≥ 110 cm	21	11				
Length of resected total bowel	< 160 cm	12	16	0.0185			
	≥ 160 cm	22	8				
Comorbidities							
Diabetes	( +)	11	6	1.0			
	(−)	43	22				
Hypertension	( +)	27	14	1.0			
	(−)	27	14				
Hyperlipidemia	( +)	14	3	0.1525			
	(−)	40	25				
Cardiovascular surgery or PCI	( +)	22	9	0.4464			
	(−)	32	19				
Chronic heart disease	( +)	27	10	0.2177			
	(−)	27	18				
Cancer bearing state	( +)	6	6	0.2100			
	(−)	48	22				
Renal dysfunction	( +)	46	20	0.1361			
	(−)	8	8				
Renal replacement therapy	( +)	3	3	0.4057			
	(−)	51	25				
Cardiopulmonary arrest	( +)	9	6	0.5969			
	(−)	45	22				
Pre-onset catecholamine administration	( +)	30	8	0.0201			
	(−)	24	20				
Pre-onset norepinephrine dose > 0.1 μg/kg/min	( +)	18	3	0.0330			
	(−)	36	25				
Pre-onset blood purification therapy	( +)	32	10	0.0421			
	(−)	22	18				
Laboratory data							
WBC (/μL)	< 10,000	23	14	0.5227			
	≥ 10,000	31	14				
Hemoglobin (g/dL)	< 10	29	10	0.1219			
	≥ 10	25	18				
CRP (mg/dL)	< 10	25	14	0.8736			
	≥ 10	29	14				
Platelet(/μL)	< 100,000	37	7	0.0002			
	≥ 100,000	17	21		3.39	0.87–14.4	0.0792
Lactate (mmol/L)	< 5	22	19	0.0292			
	≥ 5	30	9				
PT-INR	< 1.3	21	17	0.0704			
	≥ 1.3	32	11				
APTT (sec)	< 46	18	19	0.0027	3.41	0.92–13.3	0.0670
	≥ 46	31	7				
Creatinine (mg/dl)	< 1.8	26	15	0.6414			
	≥ 1.8	28	13				
AST (U/L)	< 80	23	17	0.0979			
	≥ 80	30	10				
ALT (U/L)	< 46	23	18	0.0625			
	≥ 46	31	10				
LDH (U/L)	< 430	24	16	0.2371			
	≥ 430	29	11				
Total-bilirubin (mg/dL)	< 1.3	19	15	0.1090			
	≥ 1.3	35	13				
Direct-bilirubin (mg/dL)	< 0.6	21	17	0.0602			
	≥ 0.6	33	11				
CPK (U/L)	< 270	23	15	0.3393			
	≥ 270	29	12				
SOFA score	< 11	19	18	0.0163	1.31	0.33–5.08	0.6956
	≥ 11	31	9				

*BMI* body mass index, *CECT* contrast-enhanced computed tomography, *WBC* white blood cell count, *CRP* C-reactive protein, *PT-INR* prothrombin time international normalized ratio, *APTT* activated partial thromboplastin time, *AST* aspartate transaminase, *ALT* alanine trans aminase, *LDH* lactate dehydrogenase, *CPK* creatinine phosphokinase, *SOFA* sequential organ failure assessment score

The analysis of the factors affecting the return to social activities is presented in Table 5. Factors that were significantly associated with a higher likelihood of returning to social activities included the following: positive portal venous gas on CT, no open abdomen, no pre-onset catecholamine administration, no pre-onset blood purification therapy, platelet count < 100,000/µL, lactate level < 5 mmol/L, APTT < 46 s, and SOFA score < 11. A multivariate analysis could not be performed because of the limited sample size of the patients who returned to social activities (*n* = 9).

**Table 5:** Demographics associated with return to social activities

		Return to social activities		Univariate analysis (*P* value)
		No	Yes	
Gender	Male	46	7	0.4812
	Female	27	2	
Age (years)	< 75	38	3	0.4821
	≥ 75	35	6	
BMI (kg/m^2^)	< 21	32	2	0.2843
	≥ 21	36	7	
Diagnosis procedure	CECT	47	5	0.7179
	Intra-operative findings	26	4	
CECT findings				
Defect of intestinal wall enhancement	( +)	60	7	0.4551
	(−)	4	1	
Pneumatosis intestinalis	( +)	36	7	0.1659
	(−)	35	2	
Ascites	( +)	45	3	0.1456
	(−)	26	6	
Free air	( +)	12	1	1.0
	(−)	57	6	
Hepatic portal venous gas	( +)	14	7	0.0009
	(−)	57	2	
Laparotomy	( +)	51	8	0.4332
	(−)	22	1	
Open abdomen	( +)	30	0	0.0019
	(−)	21	8	
Length of resected small bowel	< 110 cm	22	5	0.4498
	≥ 110 cm	29	3	
Length of resected total bowel	< 160 cm	22	6	0.1384
	≥ 160 cm	28	2	
Comorbidities				
Diabetes	( +)	13	4	0.0834
	(−)	60	5	
Hypertension	( +)	34	7	0.1549
	(−)	39	2	
Hyperlipidemia	( +)	17	0	0.1924
	(−)	56	9	
Cardiovascular surgery or PCI	( +)	29	2	0.4715
	(−)	44	7	
Chronic heart disease	( +)	35	2	0.1745
	(−)	38	7	
Cancer bearing state	( +)	9	3	0.1215
	(−)	64	6	
Renal dysfunction	( +)	61	5	0.0676
	(−)	12	4	
Renal replacement therapy	( +)	6	0	1.0
	(−)	67	9	
Cardiopulmonary arrest	( +)	15	0	0.1996
	(−)	58	9	
Pre-onset catecholamine administration	( +)	37	1	0.0333
	(−)	36	8	
Pre-onset norepinephrine dose > 0.1 μg/kg/min	( +)	21	0	0.1028
	(−)	52	9	
Pre-onset blood purification therapy	( +)	41	1	0.0135
	(−)	32	8	
Laboratory data				
WBC (/μL)	< 10,000	32	5	0.7247
	≥ 10,000	41	4	
Hemoglobin (g/dL)	< 10	36	3	0.4874
	≥ 10	37	6	
CRP (mg/dL)	< 10	36	3	0.8997
	≥ 10	37	6	
Platelet(/μL)	< 100,000	44	0	0.0006
	≥ 100,000	29	9	
Lactate (mmol/L)	< 5	33	8	0.0294
	≥ 5	38	1	
PT-INR	< 1.3	31	7	0.0757
	≥ 1.3	41	2	
APTT (s)	< 46	30	7	0.0284
	≥ 46	37	1	
Creatinine (mg/dL)	< 1.8	35	6	0.4821
	≥ 1.8	38	3	
AST (U/L)	< 80	34	6	0.2633
	≥ 80	38	2	
ALT (U/L)	< 46	34	7	0.1549
	≥ 46	39	2	
LDH (U/L)	< 430	33	7	0.0568
	≥ 430	39	1	
Total-bilirubin (mg/dL)	< 1.3	28	6	0.1527
	≥ 1.3	45	3	
Direct-bilirubin (mg/dL)	< 0.6	31	7	0.0740
	≥ 0.6	42	2	
CPK (U/L)	< 270	31	7	0.0802
	≥ 270	39	2	
SOFA score	< 11	30	7	0.0251
	≥ 11	39	1	

*BMI* body mass index, *CECT* contrast-enhanced computed tomography, *WBC* white blood cell count, *CRP* C-reactive protein, *PT-INR* prothrombin time international normalized ratio, *APTT* activated partial thromboplastin time, *AST* aspartate transaminase, *ALT* alanine transaminase, *LDH* lactate dehydrogenase, *CPK* creatinine phosphokinase, *SOFA* Sequential Organ Failure Assessment Score

## Discussion

It is widely accepted that NOMI involves mesenteric ischemia without occlusion of the main mesenteric vessels caused by mesenteric vasoconstriction resulting from an imbalance between oxygen demand and supply in the intestinal tract [12]. However, there are currently no preoperative consensus diagnostic criteria, partly due to the lack of characteristic findings for various biomarkers and the difficulty of early detection using imaging [13]. An early diagnosis and therapeutic intervention are crucial to improve the survival rate of patients with NOMI. Therefore, it is not worthwhile to narrow the diagnostic criteria and increase the number of cases of “intestinal ischemia of unknown cause that is not NOMI.”

In the present study, we diagnosed all cases of mesenteric ischemia without obstruction of the main mesenteric vessels as having circulatory failure. For the preoperative diagnosis, we focused on contrast-enhanced CT findings, which are known to have a low negative predictive value for NOMI [14, 15]. Therefore, it is difficult to diagnose NOMI using CT when the loss of contrast effect on the intestinal wall is not evident. The cases diagnosed as NOMI based on contrast-enhanced CT findings in this study had already shown a decreased contrast effect on the intestinal wall, indicating some progression of the condition.

Similar to NOMI, ischemic colitis is a form of intestinal ischemia that does not involve obstruction of the main mesenteric vessels. Ischemic colitis usually presents with segmental ischemia of the large intestine and rarely extends extensively [16]. It is possible to differentiate ischemic colitis from NOMI based on the general condition and the extent of ischemia. However, in advanced necrotizing ischemic colitis, peritonitis and sepsis may occur because of necrosis, resulting in abnormal circulation. Differentiating advanced ischemic colitis from NOMI is challenging despite the different pathogenesis mechanisms. Therefore, the possibility that some patients with advanced ischemic colitis were included in this study cannot be ruled out.

In the present study, we used the GPOC score to assess the post-treatment return to society among patients with NOMI. The GPOC score has been adopted in the Utstein-style guidelines for out-of-hospital cardiopulmonary arrest (CPA) [10] and is widely used to assess brain and systemic functions following cardiac resuscitation. The application of this measure aligns with the underlying mechanism of NOMI, as this condition frequently occurs during severe conditions of hypovolemia such as CPA [9, 17]. Given the lack of NOMI-specific outcome measures, we hypothesized that the GPOC score can serve as an objective functional assessment tool. The GPOC includes CPC, a neurological assessment, and OPC, an overall assessment. Therefore, we believe that the GPOC is not solely a measure of the neurological function. Although physical strength is important for returning to social activities, neurological recovery is crucial for effective communication, which is essential for social activities. Therefore, we determined that using the GPOC as an indicator of return to social activities was appropriate. Paul et al. [9] used CPC to assess the neurological function in patients with NOMI after cardiac arrest and reported that only 4% of eligible cases achieved CPC scores of 1 or 2. In the present study, we evaluated all NOMI cases, not solely those following cardiac arrest, and found that only approximately 10% of the patients could return to social activities, with CPC or OPC scores of 1–2. Although our results were more favorable than those reported by Paul et al. [9], which were exclusively obtained after cardiac arrest, the observation that only approximately 10% of the patients could return to social activities highlights the difficulties associated with diagnosing and treating NOMI. To our knowledge, this is the first study to objectively evaluate the post-treatment return to social activities in patients with NOMI not restricted to those following cardiac arrest. Because NOMI is an emergency condition that results in a sudden referral, we could not evaluate the preoperative conditions of most patients. It is possible that patients with NOMI were already in a poor general condition before the disease, which could have been responsible for their low rate of return to social activities and not so much the disease itself. Thus, evaluating the social reintegration status after the disease, including pre-existing poor conditions, is important for considering the cost-effectiveness and future treatment strategies for this disease.

The sole independent factor contributing to in-hospital mortality in the present study was non-surgical management. Notably, 21 of 23 patients with NOMI did not undergo surgery because of their critical condition. Almost all patients for whom non-operative management was chosen had systemic conditions that contraindicated surgery, which may account for this result. Several studies have explored the use of intra-arterial or intravenous vasodilators as non-surgical treatments for NOMI [18–22]. In a prospective study of intra-arterial therapy for NOMI, Rittgerodt et al. [18] reported a high 28-day mortality rate (71.8%). However, relatively few in-hospital deaths were observed among patients whose lactate levels decreased after intravenous therapy. Furthermore, the authors acknowledged the difficulty of predicting ischemic improvement and the prognosis based on imaging findings and various markers. Although the prognosis can be predicted using a post-treatment response, pretreatment prediction using currently available diagnostic methods is challenging. In our study, we actively advocated surgical management unless intestinal necrosis was definitively ruled out. This explains the limited use of arterial injection in our patients. An early diagnosis is crucial for improving survival rates, especially in cases that can be treated with intra-arterial therapy. Therefore, establishing an early diagnostic method is desirable. Until such a method becomes available, surgical intervention may be preferable when intestinal necrosis cannot be ruled out definitively.

The univariate analysis in our study showed that patients with portal venous gas on CT had significantly fewer in-hospital mortalities and higher rates of return to social activity than patients without portal venous gas on CT. Although portal venous gas imaging has been widely recognized as a marker of intestinal ischemia or necrosis, often prompting emergency surgery [23], our findings suggest otherwise. Recently, many conditions other than intestinal necrosis have been associated with this finding [24, 25]. In addition, portal venous gas represents the early ischemic phase of intestinal ischemia before necrosis [26], which is why the findings in our study cannot rule out the possibility of early-stage intestinal ischemia.

Several limitations associated with the present study warrant mention. First, it was a retrospective study involving a small number of patients at a single institution. The small sample size also precluded the multivariate analysis of factors influencing the resumption of social activity. However, our single-institution design allowed for a detailed examination of a relatively large number of cases and facilitated long-term follow-up after the diagnosis. Second, NOMI was diagnosed by surgeons based on the imaging findings. Given the context of NOMI as an emergent condition, the diagnosis is often made by emergency physicians or surgeons rather than radiologists. Furthermore, the surgeons involved in this study had more than 10 years of experience in managing emergency abdominal cases. Therefore, we believe that they maintained a reasonable diagnostic quality. Finally, a few patients were treated with intra-arterial or intravenous vasodilators, since surgeons opted for surgery unless necrosis was completely ruled out, as previously explained. The involvement of radiologists in treatment decision-making may have increased the number of interventional radiology procedures.

In conclusion, this is the first study to assess post-treatment return to social activities among patients with NOMI. Our findings highlight the concerning reality that survivors may face prolonged dependence on medical care. Therefore, the development of new diagnostic methods and modalities is warranted to improve treatment outcomes in this cohort.
